# Evaluation of Gene Expression and the Regulatory Role of microRNAs Related to the Mitogen-Activated Protein Kinase Signaling Pathway in Human Retinal Pigment Epithelial Cells Treated With Lipopolysaccharide A and Tacrolimus

**DOI:** 10.1155/mi/8586711

**Published:** 2025-07-01

**Authors:** Aleksandra Kiełbasińska, Katarzyna Krysik, Dominika Janiszewska-Bil, Martyna Machaj, Zuzanna Lelek, Mariola Szulik, Patrycja Mickiewicz, Anita Lyssek-Boroń, Beniamin Oskar Grabarek

**Affiliations:** ^1^Department of Opthalmology, University Clinical Center Named After Prof. K. Gibiński of the Medical University of Silesia in Katowice, Katowice 40-514, Poland; ^2^Department of Ophthalmology, Faculty of Medicine, Academy of Silesia, Katowice 40-555, Poland; ^3^Department of Ophthalmology, St. Barbara Hospital, Trauma Centre, Sosnowiec 41-200, Poland; ^4^Collegium Medicum, WSB University, Dabrowa Gornicza 41-300, Poland; ^5^Departament of Cardiology and Electrotherapy, Silesian Center for Heart Diseases, Faculty of Medical Sciences in Zabrze, Medical University of Silesia, Katowice 40-555, Poland; ^6^Faculty of Medicine and Health Sciences, Andrzej Frycz Modrzewski University, Cracow 30-705, Poland

**Keywords:** lipopolysaccharide A, mitogen-activated protein kinase signaling pathway, proliferative vitreoretinopathy, retinal pigment epithelium, tacrolimus

## Abstract

**Background:** The retinal pigment epithelium (RPE) is central to retinal health and immune regulation. In diseases, such as proliferative vitreoretinopathy (PVR), dysregulated RPE function, driven by aberrant signaling pathways like mitogen-activated protein kinase (MAPK), contributes to fibrotic membrane formation and retinal detachment. Tacrolimus, an immunosuppressive agent, has shown potential to modulate signaling beyond immune cells, but its effect on MAPK signaling in RPE cells remains unclear. This study aimed to investigate the impact of tacrolimus on MAPK pathway gene expression and microRNA (miRNA)-mediated regulation in human RPE (H-RPE) cells under inflammatory conditions induced by lipopolysaccharide (LPS).

**Methods:** H-RPE cells were treated with LPS and tacrolimus, and cell viability was evaluated by 3- (4,5-Dimethylthiazol-2-yl)-2,5-diphenyltetrazolium bromide (MTT) assay. Transcriptomic profiling of 300 MAPK-related genes and corresponding miRNAs was performed using Affymetrix microarrays. Key targets were validated via quantitative reverse-transcription polymerase chain reaction (RT-qPCR) and enzyme-linked immunosorbent assay (ELISA). Gene interaction networks were analyzed with STRING.

**Results:** LPS significantly suppressed MAPK pathway gene and protein expression, including transforming growth factor beta 1 (TGF-β-1), mitogen-activated protein kinase kinase 7 (MAP2K7), mitogen-activated protein kinase 3 (MAPK3), and dual specificity phosphatase 4 (DUSP4). Tacrolimus reversed these effects in a time-dependent manner, restoring expression levels and modulating regulatory miRNAs (e.g., miR-3196, miR-27a/b-3p, miR-190a-3p, miR-149-3p). STRING analysis revealed a highly connected protein network, with MAPK3, MAPK8, and TRAF6 acting as central nodes.

**Conclusion:** Tacrolimus modulates MAPK signaling in H-RPE cells by reversing LPS-induced suppression and regulating specific miRNAs. These findings suggest a potential therapeutic role for tacrolimus in mitigating inflammatory and fibrotic responses associated with PVR.

## 1. Introduction

The retinal pigment epithelium (RPE) is a monolayer of highly specialized, pigmented cells situated between the photoreceptors and the choroid, playing an indispensable role in maintaining retinal structure and function [1–3]. In addition to its physiological duties—such as nutrient transport, waste removal, and photoreceptor outer segment phagocytosis—the RPE is a key regulator of immune and inflammatory responses within the eye [4, 5]. Aberrant RPE activation and signaling contribute to the pathogenesis of several retinal disorders, including proliferative vitreoretinopathy (PVR), a sight-threatening complication characterized by the formation of contractile, fibrotic membranes on both sides of the retina, often leading to tractional retinal detachment and surgical failure following retinal detachment repair [6, 7].

Despite extensive clinical research, the molecular mechanisms underlying PVR remain incompletely understood. Among the signaling networks implicated in PVR pathophysiology, the mitogen-activated protein kinase (MAPK) signaling pathway plays a central role [8]. This pathway comprises several major cascades—including the extracellular signal-regulated kinase (ERK), c-Jun N-terminal kinase (JNK), and p38 MAPK pathways—that transduce extracellular stimuli into specific intracellular responses [9]. Through tightly regulated phosphorylation events, MAPKs orchestrate a broad spectrum of cellular processes, such as proliferation, migration, epithelial-to-mesenchymal transition (EMT), inflammation, and apoptosis [10]. In the context of PVR, dysregulated MAPK activity has been shown to promote RPE dedifferentiation, proliferation, and fibrotic transformation, processes that collectively contribute to the formation and contraction of epiretinal membranes [11, 12].

Current therapeutic options for PVR are limited and primarily surgical, with no effective pharmacological interventions available to halt or reverse disease progression. This clinical gap underscores the urgent need to identify and characterize molecular targets that could modulate key signaling pathways involved in PVR pathogenesis [13, 14]. Tacrolimus (FK506), a calcineurin inhibitor widely used in transplant medicine for its immunosuppressive properties, has recently gained attention for its pleiotropic effects on nonimmune cells [15, 16]. Beyond its classical role in T-cell suppression, tacrolimus has demonstrated the capacity to influence various intracellular signaling networks, including MAPK pathways [17]. However, the precise impact of tacrolimus on MAPK signaling in RPE cells, particularly within the context of PVR, remains poorly defined.

Additionally, microRNAs (miRNAs)—small, endogenous noncoding ribonucleic acids (RNAs) that regulate gene expression at the posttranscriptional level—have emerged as critical modulators of MAPK signaling [18, 19]. Altered miRNA expression profiles have been linked to pathological processes in PVR, including EMT, fibrosis, and chronic inflammation, suggesting that the miRNA–MAPK axis may represent a pivotal regulatory mechanism in disease progression [18–21].

Therefore, the aim of this study was to comprehensively evaluate the effects of tacrolimus on the gene expression profile and miRNAs-mediated regulation of the MAPK signaling pathway in human retinal pigment epithelial (H-RPE) cells.

## 2. Material and Methods

### 2.1. Cell Culture

H-RPE cells (Clonetics, Cat. No. 194987) were cultured in 25 cm^2^ Nunclon-coated flasks (Nunc, Germany) using RtEBM basal medium (Lonza, Switzerland) supplemented with basic fibroblast growth factor and 2% FBS. Cells were incubated in a Direct Heat CO_2_ incubator (Thermo Fisher, USA) for 24 h post-passage. To model inflammation, cells were treated with LPS (Sigma–Aldrich, Poland) at 1, 2, or 10 µg/mL for 6, 12, or 24 h. Untreated cells served as controls. Tacrolimus (FK506, Sigma–Aldrich, Poland) was applied at 0.1, 1, 10, or 100 ng/mL under similar timeframes. A combination of 10 ng/mL tacrolimus and 1 µg/mL LPS was also tested at corresponding intervals.

### 2.2. Cytotoxicity Assay

Cell viability was assessed using the 3- (4,5-Dimethylthiazol-2-yl)-2,5-diphenyltetrazolium bromide (MTT) assay, based on the mitochondrial reduction of MTT to formazan in metabolically active cells. After 48 h, absorbance was measured at 580 nm (reference 720 nm). Results were normalized to untreated controls (set as 100%).

### 2.3. Isolation of the Total RNA

Total RNA was extracted with TRIzol (Invitrogen, USA; Cat. No. 15596026) and purified using the RNeasy Mini Kit (QIAGEN, Germany; Cat. No. 74104). DNase I treatment (Fermentas, Canada; Cat. No. 18047019) was employed to eliminate genomic DNA contamination. RNA quality was verified via 1% agarose gel electrophoresis with ethidium bromide and quantified spectrophotometrically at 260 nm.

### 2.4. Messenger RNA and miRNAs Microarray Analysis

Gene expression profiling of MAPK-related mRNAs was conducted using the Affymetrix HG-U133_A2 microarray and the GeneChip 3′ IVT PLUS Kit (Cat. No. 902416), following standardized protocols. A curated list of 300 genes linked to MAPK signaling (hsa04010) was obtained from Kyoto Encyclopedia of Genes and Genomes (KEGG) pathway (accessed March 20, 2025). After cDNA synthesis, aRNA amplification and fragmentation, hybridization was performed, and signals were scanned using the Affymetrix Gene Array Scanner 3000 7G.

For miRNA profiling, the Affymetrix GeneChip miRNA 2.0 Array was used. Analysis focused on identifying miRNAs that potentially regulate differentially expressed mRNAs. TargetScan http://www.targetscan.org/) [22] and miRanda (http://mirdb.org) [23] databases were used to predict miRNA–mRNA interactions. Targets scoring above 80 were considered highly reliable, while those below 60 required further validation [23, 24].

#### 2.4.1. Quantitative Reverse-Transcription Polymerase Chain Reaction (RT-qPCR)

Next, we performed RT-qPCR analysis for four genes that differentiated the cultures exposed to LPS and tacrolimus from the control, regardless of the duration of drug exposure using the 2^−ΔΔCt^ method. β-actin (ACTB) and Glyceraldehyde-3-phosphate dehydrogenase (GAPDH) served as reference genes. Primer sequences are listed in Table 1. Thermal cycling included: reverse transcription (45°C, 10 min), polymerase activation (95°C, 2 min), followed by 40 cycles of 95°C for 5 s, 60°C for 10 s, and 72°C for 5 s.

#### 2.4.2. Enzyme-Linked Immunosorbent Assay (ELISA) Assay

The next phase of the molecular analysis focused on evaluating changes in protein levels corresponding to selected genes identified in the transcriptomic profiling. This was performed using ELISA, following the manufacturer's instructions provided with each kit. The analysis included quantification of TGF-β-1, MAP2K7, DUSP4, and (Extracellular Signal Regulated Kinase 1)ERK1 (MAPK3) protein levels in H-RPE cell lysates. The following ELISA kits, all sourced from MyBioSource (San Diego, CA, USA), were used: Human TGF beta 1 ELISA Kit for TGF-β-1, Human MAP2K7 (Dual specificity mitogen-activated protein kinase kinase 7) ELISA Kit for MAP2K7, Human Dual Specificity Protein Phosphatase 4 ELISA Kit for DUSP4, and Human ERK1 ELISA Kit for MAPK3.

### 2.5. Statistical Analysis

All statistical analyses were performed using Transcriptome Analysis Console (TAC, Thermo Fisher Scientific) and StatPlus software. Data from biological replicates (*n* = 3), *d* were assessed for normality using the Shapiro–Wilk test. Data satisfying normality assumptions were further analyzed using one-way analysis of variance (ANOVA) to detect significant differences across multiple treatment groups. Post hoc pairwise comparisons were conducted using Scheffe's test, a conservative method that controls for type I error in multiple comparisons. A *p*-value of < 0.05 was considered statistically significant.

For microarray data, differential expression analysis was conducted using the ANOVA model integrated within TAC, applying a cutoff of fold change (FC) >|4.0| and adjusted *p*-value < 0.05 (Benjamini–Hochberg false discovery rate correction) to identify significant genes across treatment groups. Venn diagrams were constructed to visualize gene expression overlaps across time points (6, 12, and 24 h).

RT-qPCR results were analyzed using the 2^−ΔΔCt^ method, with expression levels normalized to reference genes ACTB and GAPDH. Data were reported as mean ± standard deviation (SD), and statistical comparisons among treatment groups were performed via one-way ANOVA and Scheffe's test as above.

For ELISA quantification, protein concentrations were calculated from standard curves and expressed as mean ± SD. Comparisons among the LPS-treated, tacrolimus-treated, combination-treated, and control groups were conducted using two-way ANOVA (treatment × time interaction), followed by Bonferroni correction for multiple comparisons when applicable.

Finally, protein–protein interaction (PPI) enrichment analysis was performed using the STRING v11.0 database. The statistical significance of observed interactions was evaluated against a random network of the same size, with enrichment strength reported as log_10_ (observed/expected). The PPI enrichment *p*-value (< 1.0e−16) and clustering coefficient (0.677) were used to confirm biological relevance and network modularity. Enrichment analyses also included gene ontology (GO) and KEGG pathway mapping, with FDR-adjusted *p*-values reported for biological process annotations [25].

## 3. Results

### 3.1. Results of MTT Cytotoxicity Assay

The MTT assay results (Table 2) revealed that LPS at a concentration of 1 µg/mL did not significantly affect H-RPE cell viability (*p*=0.784), whereas 2 and 10 µg/mL induced statistically significant reductions over time (*p*=0.023 and *p*=0.022, respectively), particularly when comparing 6 h to later time points. In contrast, tacrolimus at 0.1 ng/mL significantly influenced cell viability between 6 and 12 h and between 12 and 24 h (*p*=0.027), suggesting a time-dependent effect at lower concentrations. Higher concentrations of tacrolimus (1–100 ng/mL) did not result in statistically significant changes in viability. The combination of LPS and tacrolimus showed a borderline nonsignificant effect (*p*=0.0786). Based on these findings and supporting literature, LPS at 1 µg/mL and tacrolimus at 10 ng/mL were selected for further experiments as they maintained cell viability while being biologically relevant concentrations for evaluating pathway modulation [26–31].

### 3.2. Evaluation of the mRNAs Microarray Analysis

**Figure 1:**
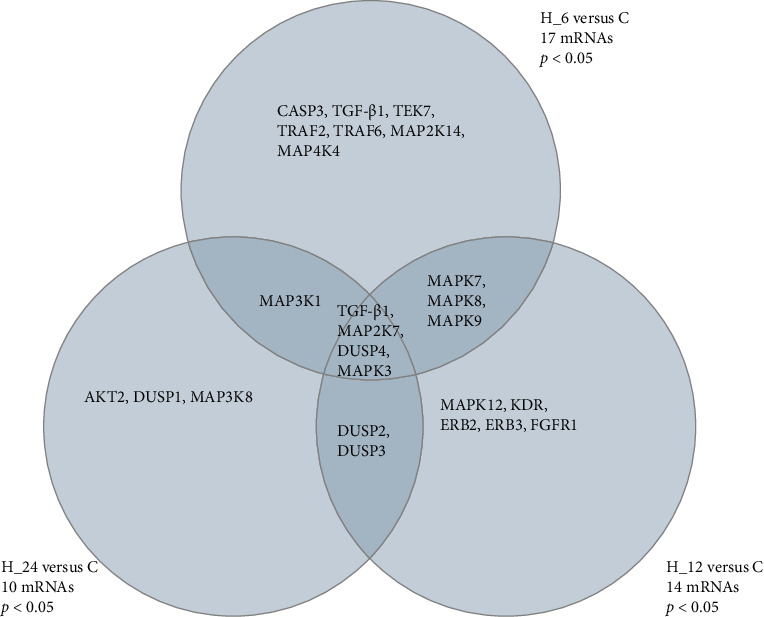
Venn diagram illustrating the overlap and specificity of differentially expressed mRNAs (|FC| > 4.0, *p* < 0.05) associated with the MAPK signaling pathway in LPS-treated H-RPE cells following tacrolimus exposure for 6, 12, and 24 h compared to untreated control. AKT2, RAC-beta serine/threonine-protein kinase; C, control cell culture; C, control culture; CASP3, Caspase-3; DUSP1, Dual specificity protein phosphatase 1; DUSP2, Dual specificity protein phosphatase 2; DUSP3, Dual specificity protein phosphatase 3; ERBB3, Receptor tyrosine-protein kinase erbB-3; FC, fold change; FGFR1, Fibroblast growth factor receptor 1; H_6, H_8, and H_24, time of exposure to the medicine; KDR, Vascular endothelial growth factor receptor 2; MAP2K14, Mitogen-activated protein kinase kinase 14; MAP2K7, Mitogen-activated protein kinase kinase 7; MAP3K1, Mitogen-activated protein kinase kinase kinase 1; MAP3K8, Mitogen-activated protein kinase kinase kinase 8; MAP4K4, Mitogen-activated protein kinase kinase kinase kinase 4; MAPK12, Mitogen-activated protein kinase 12; MAPK3, Mitogen-activated protein kinase 3; MAPK7, Mitogen-activated protein kinase 7; MAPK8, Mitogen-activated protein kinase 8; MAPK9, Mitogen-activated protein kinase 9; TEK, Angiopoietin-1 receptor (also known as TIE2); TGFB2, Transforming growth factor beta-2; TGF-β-1, Transforming growth factor beta-1; TRAF2, TNF receptor-associated factor 2; TRAF6, TNF receptor-associated factor 6.

Out of the 300 mRNAs associated with the MAPK-dependent signaling pathway selected from the KEEG database (access date 20 Mar 2025), ANOVA analysis indicated that the culture exposed to LPS, and tacrolimus differentiated 25 mRNAs with a fold change (∣FC∣) >4.00 and *p* < 0.05. A further post hoc test showed that H-RPE culture exposed to the drug for 6 h compared to control differentiated 17 mRNAs, of which 7 were specific, H-RPE culture exposed to the drug for 12 h compared to control differentiated 14 mRNAs, of which 5 were specific, and H-RPE culture exposed to the drug for 24 h compared to control differentiated 10 mRNAs, of which 3 were specific. Furthermore, statistical analysis showed that 4 mRNAs differentiated the culture exposed to the drug regardless of incubation time compared to the control culture. The diagram highlights the number of mRNAs uniquely regulated at each time point (6 h: 7 genes; 12 h: 5 genes; 24 h: 3 genes) as well as 4 mRNAs commonly regulated across all time points. A total of 25 differentially expressed mRNAs were identified based on KEGG pathway selection and ANOVA filtering (Figure 1). In turn, Table 3 shows the changes in the expression pattern of the selected 25 mRNAs (*p* < 0.05).

Microarray analysis (Table 3) revealed significant gene expression changes in H-RPE cells exposed to LPS or LPS combined with tacrolimus compared to untreated controls. LPS alone caused strong downregulation of most genes, with fold changes below –4, notably *TGF-β-1* (–9.64 ± 0.92), *MAP3K1* (–9.35 ± 0.82), and *TRAF6* (–9.40 ± 0.41). In contrast, co-treatment with LPS and tacrolimus led to substantial upregulation in nearly all genes across 6, 12, and 24 h. Some genes, such as *MAPK3*, *MAP2K7*, and *MAPK8* showed robust induction over time (e.g., MAPK3, 9.48 ± 0.55 at 24 h), suggesting a reversal of LPS-induced suppression. A few transcripts, like *TEK7* at 24 h (–6.32 ± 0.22), remained downregulated.

### 3.3. Results of RT-qPCR

**Figure 2:**
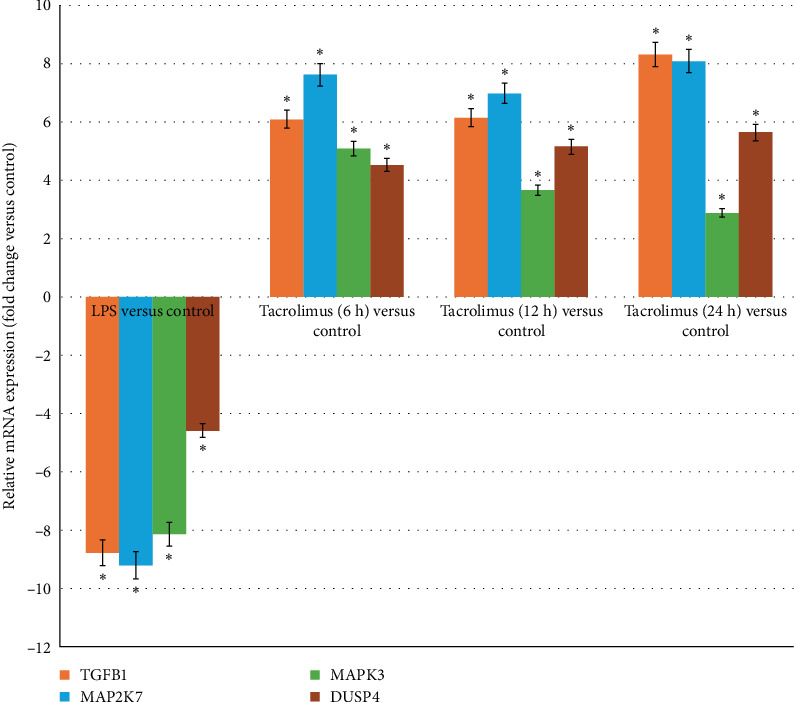
RT-qPCR analysis of four mRNAs (TGF-β1, MAP2K7, MAPK3, and DUSP4) in H-RPE cells exposed to LPS or LPS with tacrolimus at different time points (6 , 12 , and 24 h) compared to untreated control. Bars represent mean fold change (±SD) from three independent experiments. Expression was normalized to GAPDH and calculated relative to control. DUSP4, dual specificity phosphatase 4; LPS, lipopolysaccharide A; MAP2K7, mitogen-activated protein kinase kinase 7; MAPK3, mitogen-activated protein kinase 3; TGF-β1, transforming growth factor beta 1; *⁣*^*∗*^, a statistically significant difference compared to control (*p* < 0.05).

In the next stage of the study, we assessed the expression profiles of four mRNAs that consistently differentiated H-RPE cultures exposed to LPS or to LPS with tacrolimus, relative to the control group (Figure 2). RT-qPCR analysis revealed that all four genes—*TGF-β1*, *MAP2K7*, *MAPK3*, and *DUSP4*—were significantly downregulated in response to LPS alone. However, co-treatment with tacrolimus resulted in a marked upregulation of these genes, beginning at 6 h and remaining elevated at 12 and 24 h. These findings suggest that tacrolimus counteracts the LPS-induced transcriptional repression and promotes sustained gene activation over time. The gene expression patterns observed in RT-qPCR closely mirrored the results obtained from the oligonucleotide microarray analysis, confirming the reliability of the data.

### 3.4. Expression Profile of miRNAs Potentially Regulated Selected mRNA Related to TGF-β1, MAP2K7, MAPK3, and DUSP4 in H-RPE Cells Treated With LPS and Tacrolimus in Comparison to a Control Culture

The integrated mRNA–miRNA expression analysis revealed distinct regulatory interactions (Table 4; *p* < 0.05). TGF-β1 mRNA was positively correlated with hsa-miR-3196, showing progressive upregulation across all treatment conditions (log_2_FC up to 2.91 ± 0.91, *p* < 0.05). MAP2K7 was targeted by both hsa-miR-27b-3p and hsa-miR-27a-3p with high target scores (83), showing a notable increase in expression despite the negative log fold change of miR-27a-3p under LPS (–3.24 ± 0.67), suggesting miRNA-mediated repression under LPS and relief following tacrolimus treatment. MAPK3 expression increased over time, while its targeting miRNA, hsa-miR-190a-3p, was downregulated under LPS (–2.11 ± 0.19), indicating inverse regulation. *DUSP4* displayed consistent downregulation after tacrolimus exposure, correlating with increased levels of hsa-miR-149-3p, suggesting posttranscriptional silencing. All changes were statistically significant (*p* < 0.05).

### 3.5. Results of ELISA Assay

Table 5 presents changes in the concentration of selected proteins in H-RPE cell cultures exposed to LPS, tacrolimus (at 6, 12, and 24 h), and a control culture. Exposure to LPS alone resulted in a significant reduction in TGF-β-1, MAP2K7, and MAPK3 (ERK1) levels compared to the control (*p* < 0.05), while DUSP4 showed a significant increase. In contrast, treatment with tacrolimus following LPS exposure led to a marked and time-dependent increase in TGF-β-1 levels, from 765.10 ± 15.21 pg/mL at 6 h to 1911.19 ± 21.71 pg/mL at 24 h. MAP2K7 and MAPK3 concentrations also rose significantly under tacrolimus treatment at all time points. Conversely, DUSP4 levels, initially elevated in response to LPS, progressively declined after tacrolimus exposure, though remaining above control values.

### 3.6. Result of STRING Network Analysis

**Figure 3:**
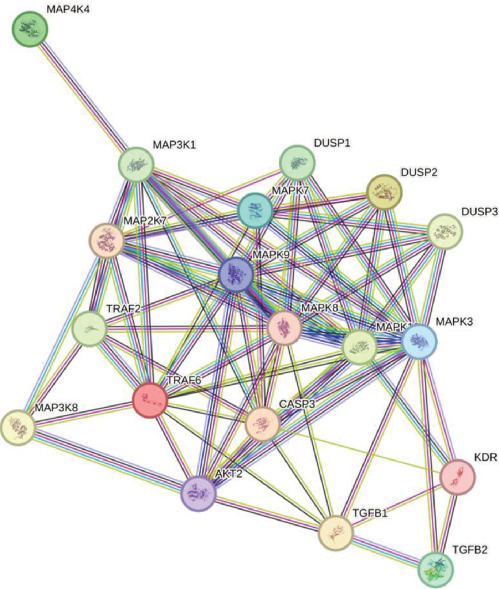
Protein interaction network for MAPK differentiation-related genes generated using the STRING database.

The STRING network analysis of 19 proteins involved in the MAPK signaling pathway in H-RPE cells demonstrates a highly interconnected system with 78 PPIs, significantly exceeding the 11 edges expected by random chance (PPI enrichment *p*-value <1.0e-16). This strong enrichment underscores the biological relevance and coordination of these proteins within a functional signaling module. The average node degree of 8.21 indicates that each protein interacts with multiple partners, suggesting that key regulators, such as MAPK8 (JNK1), MAPK9 (JNK2), MAPK3 (ERK1), and MAPK14 (p38α) serve as central hubs in transmitting extracellular signals to intracellular responses. Proteins, such as TRAF6 and MAP3K1 appear to act as upstream adaptors or kinases, activating the MAPK cascade in response to stress or pro-fibrotic stimuli. Additionally, dual-specificity phosphatases (DUSPs), such as DUSP1, DUSP2, and DUSP3, are present in the network and serve as negative regulators, modulating pathway output through dephosphorylation of MAPKs. The clustering coefficient of 0.677 reflects a high degree of modularity, consistent with the existence of tightly regulated signaling subnetworks that could correspond to ERK, JNK, and p38 branches (Figure 3).

## 4. Discussion

Following a retinal tear or detachment, RPE cells are displaced into the vitreous cavity, where they encounter a microenvironment rich in pro-inflammatory cytokines and growth factors [2, 32]. This exposure initiates a complex autocrine and paracrine signaling loop, wherein RPE cells begin to secrete additional mediators that amplify their own activation and influence neighboring cells. This dynamic interaction contributes to the initiation and progression of PVR [33].

In the PVR milieu, several cell types participate in the fibrotic response, including fibroblasts, fibrous astrocytes, macrophages, and particularly RPE-derived myofibroblasts. Among these, RPE cells and transdifferentiated myofibroblasts are considered the dominant cellular contributors to the formation and contraction of fibrotic membranes on or beneath the retina [34]. The pathophysiology of PVR is characterized by the proliferation and migration of these cells, excessive extracellular matrix (ECM) deposition, formation of epiretinal membranes, and their eventual contraction, which can culminate in recurrent retinal detachment and vision loss [35, 36].

Recent research has underscored the pivotal role of inflammatory mediators—especially cytokines and growth factors—in orchestrating these events. Elevated levels of these biomolecules have been consistently detected in the vitreous humor and subretinal fluid of patients with PVR, supporting the concept of an inflammation-driven fibrotic mechanism [8, 37]. This has led to the formulation of the “growth factor and cytokine hypothesis,” which posits that abnormal expression of these signaling molecules is a driving force behind the development and progression of PVR [38].

Interestingly, a progressive reduction in the number of significantly regulated mRNAs was observed in H-RPE cells treated with tacrolimus over extended incubation periods. This temporal decline in transcriptional responsiveness likely reflects the diminishing biological activity of tacrolimus, which may be attributable to its intracellular pharmacokinetics. Tacrolimus is known to have a relatively short intracellular half-life and undergoes rapid metabolic inactivation, primarily via cytochrome P450 3A isoenzymes, such as CYP3A4 and CYP3A5 [39, 40]. Additionally, the drug's sequestration in intracellular organelles, including lysosomes and the endoplasmic reticulum, may further limit its duration of action within nonimmune cell types. These findings underscore the importance of maintaining effective intracellular concentrations to preserve therapeutic efficacy, potentially through repeated administration or controlled-release delivery systems, particularly in chronic inflammatory or fibrotic conditions, such as PVR.

Importantly, the concentration of tacrolimus applied in our experiments (10 ng/mL) was selected based on prior cytotoxicity screening using the MTT assay, which confirmed no adverse effects on cell viability across all tested time points. Thus, the transcriptomic and proteomic changes observed in H-RPE cells can be attributed to the specific pharmacodynamic activity of tacrolimus, rather than nonspecific cytotoxicity. This supports its profile as a biologically active yet well-tolerated agent, capable of modulating key signaling pathways, such as MAPK in the RPE under inflammatory stress.

Our findings demonstrate that tacrolimus modulates MAPK signaling in H-RPE cells through a multilayered regulatory architecture involving transcriptional, posttranscriptional, and translational control mechanisms. Notably, the expression of *TGF-β-1*, *MAP2K7*, *MAPK3*, and *DUSP4* was not only significantly altered at the mRNA level but also mirrored at the protein level in a time-dependent manner, validating the robustness of these targets as effectors of tacrolimus action. The observed changes in gene and protein expression were tightly interwoven with alterations in the expression of specific regulatory miRNAs, suggesting a functional mRNA–miRNA–protein axis. This axis holds potential for both mechanistic understanding and translational biomarker development in the context of PVR.

These four mRNAs—*TGF-β1*, *MAP2K7*, *MAPK3*, and *DUSP4*—were prioritized for further analysis not only due to their involvement in key branches of the MAPK signaling cascade but also because they consistently differentiated LPS- and tacrolimus-exposed H-RPE cells from controls across all incubation time points. This temporal consistency suggests their potential utility as robust molecular indicators of tacrolimus activity. Furthermore, their expression was accompanied by coordinated changes in specific miRNAs, indicating that these mRNAs may serve as central nodes in a regulatory miRNA–mRNA network responsive to tacrolimus. Taken together, these features point to their value as candidate biomarkers for monitoring the anti-inflammatory and anti-fibrotic effects of tacrolimus in RPE cells and potentially in the broader context of PVR-related fibrosis.

*TGF-β*-1 was among the most dramatically downregulated genes and proteins following LPS treatment, underscoring the acute suppression of anti-inflammatory or homeostatic TGF-β signaling during retinal inflammation [12, 40, 41]. However, upon tacrolimus co-treatment, both TGF-β-1 mRNA and protein levels were progressively restored, achieving a > 4.5-fold increase at 24 h post-exposure. This was accompanied by a consistent and time-dependent upregulation of miR-3196, which is positively correlated with TGF-β1. Although the canonical role of miRNAs involves posttranscriptional repression via mRNA degradation or translational inhibition, emerging evidence suggests that certain miRNAs can act through noncanonical, enhancer-like mechanisms, stabilizing target mRNAs or even facilitating translation in a context-dependent manner [42]. In particular, miR-3196 displayed a positive correlation with mRNA TGF-β-1 and protein levels across all time points following tacrolimus treatment. This atypical pattern may reflect the action of miRNA-mediated RNA activation (RNAa), a mechanism wherein miRNAs interact with AU-rich elements or specific binding sites in the 5′ or 3′ UTR to recruit RNA-binding proteins or translational machinery, thereby enhancing transcript stability and translation efficiency [43, 44]. Such regulatory behavior has been documented for miRNAs, such as miR-10a and miR-346 [45] in other systems, and may suggest a context-specific coactivator role for miR-3196 in RPE cells under tacrolimus exposure.

The unique upregulation of miR-3196 in parallel with TGF-β-1 may reflect a feedback amplification loop intended to restore cellular equilibrium or may represent a cell-type-specific epiphenomenon unique to RPE. While specific studies directly linking miR-3196 to TGF-β1 regulation in RPE cells are limited, the role of TGF-β in inducing EMT and fibrosis is well-documented [46]. For instance, TGF-β1 stimulation in RPE cells increases α-SMA expression, cell migration, and contractility—hallmarks of EMT—mediated by TAK1 signaling [47, 48]. Additionally, TGF-β plays a prominent role in EMT during development and wound healing, as well as in pathological conditions like fibrosis and cancer [49–51]. Furthermore, studies have shown that TGF-β signaling pathways are involved in the development of diabetic retinopathy [52], with miR-200a-3p modulating TGF-β2 expression in human RPE cells [53]. These findings underscore the complexity of TGF-β signaling in RPE cells and its potential implications in PVR progression [54]. Further research is warranted to elucidate the specific interactions between miR-3196 and TGF-β1, which could provide valuable insights into therapeutic strategies targeting TGF-β1 in retinal diseases.

*MAP2K7*, a mitogen-activated protein kinase kinase upstream of the JNK signaling axis, demonstrated significant transcriptional repression following LPS exposure, an effect that was notably reversed upon treatment with tacrolimus. At the posttranscriptional level, differential expression of two closely related miRNAs—miR-27a-3p and miR-27b-3p—was observed, suggesting complex miRNA-mediated regulatory dynamics. Specifically, LPS exposure resulted in a pronounced upregulation of miR-27a-3p (log_2_FC = –3.24), consistent with canonical miRNA-induced gene silencing mechanisms targeting *MAP2K7* [55–57]. In contrast, tacrolimus administration suppressed miR-27a-3p levels while concurrently enhancing the expression of miR-27b-3p, accompanied by a parallel restoration of *MAP2K7* mRNA and protein levels. This divergent miRNA response implies selective modulation of miRNA subsets by tacrolimus, potentially through differential transcriptional regulation, stability, or turnover of homologous miRNA species.

Such asymmetric regulation of paralogous miRNAs within the same seed family—particularly within ocular contexts—is rarely documented, and may reflect differences in miRNA–target interaction kinetics, secondary mRNA structure, or RNA-binding protein recruitment in inflamed versus immunomodulated RPE microenvironments [58]. Functionally, *MAP2K7*serves as a pivotal regulator of JNK [59]-driven pathways implicated in EMT, pro-inflammatory signaling, and apoptosis—all key processes in the pathogenesis of PVR [48]. The tacrolimus-mediated rescue of *MAP2K7* thus represents not only a mechanistic checkpoint in the modulation of JNK signaling but also a candidate biomarker reflecting therapeutic efficacy.

Similarly, *MAPK3* (ERK1), a core component of the ERK/MAPK cascade, plays an essential role in cellular proliferation, stress adaptation, and survival of RPE cells [60, 61]. LPS treatment resulted in downregulation of *MAPK3* expression at both transcript and protein levels, coinciding with a significant increase in miR-190a-3p expression, supporting a model of miRNA-mediated repression [62]. Tacrolimus treatment reversed this effect, restoring *MAPK3* expression in conjunction with a marked reduction in miR-190a-3p levels, consistent with translational derepression. This inverse correlation underscores a functional interaction wherein miR-190a-3p may directly target the 3′ untranslated region of *MAPK3*mRNA, attenuating its translation under inflammatory conditions [63].

Restoration of *MAPK3* expression likely contributes to tacrolimus-induced cytoprotection, as ERK1/2 signaling has been well established to promote RPE cell viability, inhibit apoptotic signaling cascades, and counteract oxidative stress [64, 65]. Therefore, the miR-190a-3p–*MAPK3* regulatory axis may serve as a molecular sensor of the cellular switch from stress-induced apoptosis toward survival and repair, providing translational relevance as a dynamic marker for pharmacological intervention in retinal degenerative processes [66].

In contrast to the activation of kinase signaling components, *DUSP4* also known as MAP kinase phosphatase-2 (MKP-2)—a dual-specificity phosphatase that dephosphorylates and inactivates MAPKs [67]—exhibited an inverse regulatory profile. LPS exposure led to increased *DUSP4*expression, potentially representing a negative feedback mechanism to attenuate MAPK hyperactivation [68, 69]. Following tacrolimus administration, *DUSP4* levels progressively declined, although they remained above baseline. This downregulation correlated with upregulation of miR-149-3p, suggesting miRNA-mediated posttranscriptional repression [70]. Given the role of *DUSP4* in terminating MAPK signaling, its partial suppression under tacrolimus may reflect a controlled dampening of inhibitory signaling to permit balanced MAPK activation. This regulatory fine-tuning further emphasizes the modulatory—not merely suppressive—nature of tacrolimus within the context of RPE cell stress signaling.

DUSP4 plays a critical role in terminating MAPK signaling by dephosphorylating both threonine and tyrosine residues on activated ERK1/2, JNK, and p38 kinases. This deactivation mechanism makes DUSP4 a key negative feedback regulator within the MAPK cascade, acting to fine-tune signal amplitude and duration in response to stress or cytokine exposure [68, 69].

In our study, exposure of H-RPE cells to LPS led to significant upregulation of DUSP4, consistent with prior observations that pro-inflammatory stimuli often induce MAPK pathway feedback inhibitors in an attempt to restrain excessive activation [10, 55]. This compensatory increase in DUSP4 may reflect a cellular effort to limit MAPK-driven pro-inflammatory and proapoptotic signaling.

Interestingly, treatment with tacrolimus after LPS exposure resulted in a progressive downregulation of DUSP4 expression, both at the mRNA and protein levels. This inverse relationship suggests that tacrolimus may actively suppress DUSP4 expression to enable reactivation of ERK and JNK signaling arms, which are essential for cell survival and antiapoptotic functions in RPE cells under oxidative or inflammatory stress [67, 70, 71].

Mechanistically, this downregulation was paralleled by an upregulation of miR-149-3p, a microRNA predicted to target the DUSP4 3′UTR, supporting the hypothesis that tacrolimus exerts posttranscriptional control over MAPK signaling via miRNA modulation. This is consistent with emerging models in which tacrolimus does not globally suppress inflammation, but rather selectively modulates signaling nodes to restore homeostasis [42, 71].

Therefore, the regulation of DUSP4 by LPS and tacrolimus appears to represent a biphasic signaling adjustment: an initial increase under LPS to restrict overactive MAPK signaling, followed by a partial decrease under tacrolimus to permit beneficial MAPK pathway reactivation. This dynamic supports the concept of MAPK pathway tuning, rather than binary inhibition or activation, as a therapeutic strategy [67, 68]. Such modulation allows for controlled propagation of cytoprotective MAPK activity while preventing chronic overactivation linked to fibrosis and apoptosis.

These results suggest that tacrolimus not only reactivates beneficial MAPK pathways (e.g., ERK and JNK arms) but also tempers the overactivation of negative feedback regulators, such as DUSP4, allowing for controlled signal propagation [71]. Importantly, the incomplete suppression of DUSP4 may reflect a protective mechanism to prevent pathway hyperactivation, aligning with the concept of “MAPK tuning” rather than binary on/off signaling [68, 69].

Collectively, these results provide a compelling view of tacrolimus as a modulator of signaling homeostasis in inflamed RPE cells, capable of restoring functional MAPK signaling not only through transcriptional reactivation but also by selectively regulating the miRNA landscape. The coherent upregulation of TGF-β1, MAP2K7, and MAPK3, alongside tailored suppression of DUSP4, supports a systems-level recalibration of the intracellular signaling environment under tacrolimus influence. Moreover, the synchronized changes at mRNA, protein, and miRNA levels reinforce the validity of these molecules as integrated biomarkers of tacrolimus efficacy and PVR progression.

## 5. Conclusion

This study provides novel insights into the molecular effects of tacrolimus on H-RPE cells under inflammatory conditions. We demonstrated that tacrolimus reverses LPS-induced suppression of key genes within the MAPK signaling pathway—specifically TGF-β1, MAP2K7, MAPK3, and DUSP4—at both transcript and protein levels. These effects were intricately modulated by specific miRNAs, including miR-3196, miR-27a/b-3p, miR-190a-3p, and miR-149-3p, indicating a multilayered regulatory network involving transcriptional and posttranscriptional mechanisms.

The coordinated expression patterns observed in this study suggest that these genes and their regulatory miRNAs function as an integrated molecular axis responsive to tacrolimus. While their consistent regulation across time points supports their potential utility as supplementary molecular markers for assessing anti-inflammatory and anti-fibrotic responses in RPE cells, further in vivo studies are required to validate their biomarker relevance and therapeutic applicability in clinical contexts. These findings provide a foundation for future investigations into tacrolimus as a modulator of MAPK-driven pathological signaling in retinal disease.

## Figures and Tables

**Table 1:** Primers sequences used to RT-qPCR.

mRNA	Starter	Sequence
TGF-β1	Forward	5′-TGAACCGGCCTTTCCTGCTTCTCATG-3′
	Reverse	5′-GCGGAAGTCAATGTACAGCTGCCGC-3′

MAP2K7	Forward	5′-GACAGTTTCCCTACAAGAAC-3′
	Reverse	5′-CCTGTGATCTTTAGTAAGGC-3′

MAPK3	Forward	5′-AAGATCAGCCCCTTCGAACA-3′
	Reverse	5′-CCATCAGGTCCTGCACAATG-3′

DUSP4	Forward	5′-GACCGGCAAAAATACACGGG-3′
	Reverse	5′-GAACCTAGGATGTAGCCCGC-3′

ACTB	Forward	5′-TCACCCACACTGTGCCCATCTACGA-3′
	Reverse	5′-CAGCGGAACCGCTCATTGCCAATGG-3′

GAPDH	Forward	5′- GGTGAAGGTCGGAGTCAACGGA-3′
	Reverse	5′- GAGGGATCTCGCTCCTGGAAGA-3′

Abbreviations: ACTB, actin beta; DUSP4, dual specificity phosphatase 4; GAPDH, glyceraldehyde-3-phosphate dehydrogenase; MAP2K7, mitogen-activated protein kinase kinase 7; MAPK3, mitogen-activated protein kinase 3; TGF-β1, transforming growth factor beta 1.

**Table 2:** Results of cytotoxicity test.

Treatment	Dose	Time (h)	Cell viability (%)	*p*-Value
LPS	1 µg/mL	6	94.54 ± 1.34	0.784
		12	94.98 ± 0.3	
		24	94.34 ± 0.81	

LPS	2 µg/mL	6	96.73 ± 0.45	0.023 (6 h vs. 12 h, 6 h vs. 24 h)
		12	94.96 ± 0.39	
		24	94.5 ± 0.26	

LPS	10 µg/mL	6	95.33 ± 0.23	0.022 (6 h vs. 24 h)
		12	94.1 ± 0.5	
		24	93.2 ± 0.76	

Tacrolimus	0.1 ng/mL	6	97.04 ± 0.18	0.0027 (6 h vs. 12 h, 12 h vs. 24 h)
		12	98.43 ± 0.38	
		24	96.62 ± 0.33	

Tacrolimus	1 ng/mL	6	100.78 ± 1.32	0.077
		12	97.51 ± 0.48	
		24	96.8 ± 0.5	

Tacrolimus	10 ng/mL	6	104.04 ± 0.46	0.073
		12	103.6 ± 0.37	
		24	102.28 ± 0.92	

Tacrolimus	100 ng/mL	6	99.99 ± 0.87	0.052
		12	99.03 ± 0.11	
		24	97.81 ± 0.81	

LPS + Tacrolimus	1 µg/mL+ 10 ng/mL	6	95.5 ± 0.47	0.0786
		12	95.45 ± 0.41	
		24	94.22 ± 0.64	

Abbreviation: LPS, lipopolysaccharide A.

**Table 3:** Microarray profile of selected mRNAs in H-RPE cell culture exposed to LPS or LPS and tacrolimus in comparison with a control cell culture.

mRNA	LPS versus C	H_6 versus C	H_12 versus C	H_24 versus C
AKT2	−6.25 ± 0.76	7.59 ± 0.24	4.35 ± 0.64	8.25 ± 0.97
DUSP1	−8.99 ± 0.26	5.10 ± 0.57	6.59 ± 0.65	4.84 ± 0.43
MAP3K8	−6.74 ± 0.28	7.09 ± 0.14	7.65 ± 0.16	9.69 ± 0.83
DUSP2	−5.83 ± 0.72	6.64 ± 0.55	4.21 ± 0.33	7.98 ± 0.57
DUSP3	−7.28 ± 0.97	8.65 ± 0.91	7.59 ± 0.18	5.18 ± 0.39
DUSP4	−5.16 ± 0.19	4.56 ± 0.19	5.07 ± 0.89	5.12 ± 1.01
MAPK12	−6.33 ± 0.85	6.14 ± 0.59	4.85 ± 0.17	9.92 ± 0.28
KDR	−4.03 ± 0.74	8.37 ± 0.17	6.15 ± 0.88	7.74 ± 0.16
ERB2	−4.16 ± 0.99	5.56 ± 0.91	6.43 ± 0.87	6.71 ± 0.13
ERB3	−5.87 ± 0.76	7.83 ± 0.52	4.72 ± 0.78	7.37 ± 0.54
FGFR1	−7.14 ± 0.12	4.65 ± 0.67	5.89 ± 0.92	5.50 ± 0.78
MAPK7	−5.37 ± 0.36	4.97 ± 0.83	7.80 ± 0.82	5.12 ± 0.59
MAPK8	−8.84 ± 0.39	4.66 ± 0.48	8.91 ± 0.11	7.06 ± 0.30
MAPK9	−4.72 ± 0.95	5.94 ± 0.73	6.18 ± 0.97	5.51 ± 0.37
CASP3	5.71 ± 0.65	2.56 ± 0.41	+3.11 ± 0.43	4.79 ± 0.32
TGFB2	−8.03 ± 0.31	3.50 ± 0.39	5.14 ± 0.11	5.88 ± 0.27
TEK7	−4.24 ± 0.71	4.10 ± 0.30	7.87 ± 0.72	−6.32 ± 0.22
TRAF2	6.05 ± 0.93	9.26 ± 0.69	−8.90 ± 0.58	5.45 ± 0.91
TRAF6	−9.40 ± 0.41	6.10 ± 0.91	932 ± 0.68	4.50 ± 0.91
MAP2K14	−7.64 ± 0.19	7.98 ± 0.24	7.29 ± 0.69	5.35 ± 0.31
MAP4K4	−5.95 ± 0.68	9.10 ± 0.61	4.56 ± 0.34	5.46 ± 0.45
MAP3K1	−9.35 ± 0.82	7.02 ± 0.54	5.17 ± 0.35	4.15 ± 0.26
TGF-β-1	−9.64 ± 0.92	6.22 ± 0.94	6.57 ± 0.97	9.12 ± 0.45
MAP2K7	−9.11 ± 0.25	7.34 ± 0.73	7.42 ± 0.65	9.94 ± 0.57
MAPK3 (ERK1)	−9.26 ± 0.73	5.12 ± 0.34	3.68 ± 0.31	2.90 ± 0.41

*Note*: AKT2, RAC-beta serine/threonine-protein kinase; C, control cell culture; C: control culture; H_6, H_8, and H_24, time of exposure to the medicine; KDR, vascular endothelial growth factor receptor 2; TEK, angiopoietin-1 receptor (also known as TIE2).

Abbreviations: CASP3, caspase-3; DUSP1, dual specificity protein phosphatase 1; DUSP2, dual specificity protein phosphatase 2; DUSP3, dual specificity protein phosphatase 3; DUSP4, dual specificity protein phosphatase 4; ERBB2, receptor tyrosine-protein kinase erbB-2; ERBB3, receptor tyrosine-protein kinase erbB-3; FC, fold change; FGFR1, fibroblast growth factor receptor 1; LPS, lipopolysaccharide A; MAP2K14, mitogen-activated protein kinase kinase 14; MAP2K7, mitogen-activated protein kinase kinase 7; MAP3K1, mitogen-activated protein kinase kinase kinase 1; MAP3K8, mitogen-activated protein kinase kinase kinase 8; MAP4K4, mitogen-activated protein kinase kinase kinase kinase 4; MAPK12, mitogen-activated protein kinase 12; MAPK3, mitogen-activated protein kinase 3; MAPK7, mitogen-activated protein kinase 7; MAPK8, mitogen-activated protein kinase 8; MAPK9, mitogen-activated protein kinase 9; TGFB2, transforming growth factor beta-2; TGF-β-1, transforming growth factor beta-1; TRAF2, TNF receptor-associated factor 2; TRAF6, TNF receptor-associated factor 6.

**Table 4:** Differential expression of selected mRNAs and their regulatory miRNAs in H-RPE cells treated with LPS or LPS and tacrolimus compared to control.

mRNA	miRNA	Target score	Log FC H_LPS versus C	Log FC (H_6 versus H_C)	log_2_FC (H_12 versus H_C)	log_2_FC (H_24 versus H_C)	*p*-Value
TGF-β-1	hsa-miR-3196	80	1.43 ± 0.14	2.19 ± 0.56	2.87 ± 0.77	2.91 ± 0.91	< 0.05

MAP2K7	hsa-miR-27b-3p	83	2.12 ± 0.13	3.14 ± 0.76	3.98 ± 0.91	+ 3.01 ± 0.87	< 0.05
	hsa-miR-27a-3p	83	− 3.24 ± 0.67	2.15 ± 0.19	2.98 ± 0.76	2.19 ± 0.46	< 0.05

MAPK3 (ERK1)	hsa-miR-190a-3p	89	− 2.11 ± 0.19	2.51 ± 0.86	2.12 ± 0.55	2.34 ± 0.61	< 0.05

DUSP4	hsa-miR-149-3p	97	1.11 ± 0.17	− 1.87 ± 0.53	− 2.18 ± 0.43	− 2.91 ± 0.72	< 0.05

*Note*: C, control cell culture; C: control culture; H_6, H_8, and H_24, time of exposure to the medicine

Abbreviations: DUSP4, dual specificity phosphatase 4; FC, fold change; LPS, lipopolysaccharide A; MAP2K7, mitogen-activated protein kinase kinase 7; MAPK3, mitogen-activated protein kinase 3; TGF-β-1, transforming growth factor beta 1.

**Table 5:** Changes in the concentration of selected proteins in cell culture exposed to LPS, tacrolimus, and a control culture.

Protein	H_C	LPS	H_6	H_12	H_24
TGF-β-1 (pg/mL)	398.19 ± 23.45	298.91 ± 3.45*⁣*^*∗*^	765.10 ± 15.21*⁣*^*∗*^	1291.98 ± 12.74*⁣*^*∗*^	1911.19 ± 21.71
MAP2K7	2.89 ± 0.16	1.56 ± 0.19*⁣*^*∗*^	7.87 ± 0.91*⁣*^*∗*^	7.98 ± 1.01*⁣*^*∗*^	8.12 ± 1.09*⁣*^*∗*^
MAPK3 (ERK1) (ng/mL)	3.76 ± 0.68	1.12 ± 0.87*⁣*^*∗*^	5.65 ± 0.81*⁣*^*∗*^	7.89 ± 0.91*⁣*^*∗*^	7.65 ± 0.43*⁣*^*∗*^
DUSP4 (ng/mL)	1.87 ± 0.11*⁣*^*∗*^	5.19 ± 0.68*⁣*^*∗*^	4.65 ± 0.76*⁣*^*∗*^	3.33 ± 0.42*⁣*^*∗*^	3.01 ± 0.17*⁣*^*∗*^

*Note*: C, control cell culture; C: control culture; H_6, H_8, and H_24, time of exposure to the medicine.

Abbreviations: DUSP4, dual specificity phosphatase 4; LPS, lipopolysaccharide A; MAP2K7, mitogen-activated protein kinase kinase 7; MAPK3, mitogen-activated protein kinase 3; TGF-β1, transforming growth factor beta 1.

*⁣* ^*∗*^Statistically significance versus C.

## Data Availability

The data that support the findings of this study are available from the corresponding author upon reasonable request.
